# Safety and Efficacy of Remimazolam for General Anesthesia in Elderly Patients Undergoing Transurethral Surgery

**DOI:** 10.1002/hsr2.71756

**Published:** 2026-01-16

**Authors:** Zhi Cheng, Jiachi Li, Ying Zhang, Guangrong Dai

**Affiliations:** ^1^ Department of Anesthesiology The Affiliated Lianyungang Hospital of Xuzhou Medical University Lianyungang Lianyungang China

**Keywords:** propofol, remimazolam tosylate, safety and efficacy, transurethral surgery

## Abstract

**Background and Aims:**

Remimazolam, a novel benzodiazepine, acts on the GABA receptor and is widely used for procedural sedation. To date, there are relatively few articles comparing remimazolam to propofol for general anesthesia in elderly urology patients. We compared the effectiveness and safety of remimazolam with flumazenil compared to propofol for general anesthesia in patients undergoing geriatric transurethral surgery.

**Methods:**

Ninety‐six patients were randomly assigned to the propofol group (Group P) and the remimazolam group (Group R) from November 2021 to May 2022. The primary endpoints were the occurrence of hypotension during general anesthesia. The secondary outcomes included the success rate of sedation, intraoperative haemodynamic indices, perioperative inflammatory factor levels, quality of postoperative recovery, duration of extubation and PACU stay, and the incidence of adverse reactions in both groups.

**Results:**

Occurrence rate and duration of hypotension were lower in the remimazolam group (50% vs. 69.2%, *p* = 0.096; 0 [0,10] vs. 7.5 [1.24, 25]min, *p* = 0.008). Both groups could provide satisfactory sedation.

Remimazolam had less impact on hemodynamics during induction and intraoperative process. The remimazolam group had lower QoR‐15 scores on the first postoperative day, reflected in physical comfort and emotional state. The levels of IL‐6 and TNF‐α increased after surgery. In terms of adverse reactions, the incidence of nausea, vomiting, and hiccups in the remimazolam group was higher. Although the extubation time after antagonism in the remimazolam group was shorter, the time to reach the standard of leaving the post‐anesthesia care unit in the remimazolam group was longer than in the propofol group.

**Conclusion:**

Remimazolam is both safe and effective for transurethral surgery in elderly patients. However, we need to be mindful of the need for monitoring after extubation and the possibility of a temporary reduction in the quality of recovery.

## 1 Introduction

With the aging of the population, the incidence of urological diseases among elderly patients is on the rise. Relevant studies have shown that the incidence in the elderly population can range from 25% to 50%. Additionally, a prior study found that the incidence of urinary tract infections increased significantly with age, and more than half developed self‐reported physician‐diagnosed urinary tract infections by age 60 [1].

Advancements in urological surgical techniques and fiberoptic endoscopic devices have enabled a growing number of elderly patients to receive transurethral endoscopic interventions, aiming to improve urological symptoms and quality of life. However, most patients undergoing such surgery are elderly and frequently suffer from various preexisting comorbidities. Maintaining stable blood pressure in geriatric urology is crucial for renal function protection and postoperative recovery. The intraoperative lithotomy position, extensive use of flushing fluid, application of shock wave and laser, are prone to hypotension and other adverse events during the perioperative period. Therefore, a smooth anesthesia induction and maintenance process is particularly essential.

Before the development of remimazolam, propofol had been the primary intravenous anesthetic in clinical anesthesia, despite its cardiovascular and respiratory depressive effects. Remimazolam is a new water‐soluble benzodiazepine sedative, which has the characteristics of quick onset, quick recovery, and less impact on circulation and respiration [2]. For elderly patients in urology, the selection of anesthetic drugs should take into account safety, effectiveness, and postoperative rehabilitation. Remimazolam has advantages such as minimal impact on circulation and respiration, metabolism not dependent on liver and kidney functions. As the prevalence of urological disorders is high in the elderly population, there is a lack of studies on the use of remimazolam in general anesthesia for elderly patients. Given this, we hypothesized that remimazolam might be an ideal intravenous anesthetic for elderly patients undergoing transurethral surgery. This study aimed to evaluate the effects of remimazolam and propofol on the general anesthesia process and the quality of early recovery in elderly patients undergoing transurethral surgery.

## 2 Materials and Methods

The study included a total of 96 patients who underwent transurethral surgery at the Affiliated Lianyungang Hospital of Xuzhou Medical University from November 2021 to May 2022. The patients were divided into the propofol group (Group P) and the remimazolam group (Group R) by a random number table generated by SPSS. The grouping information was sealed within opaque envelopes to uphold the principle of blinding. The patients and postoperative follow‐up were blinded to the trial assignment. The anesthesiologists were not blinded to the group assignment due to the huge discrepancy in appearance between propofol and remimazolam.

### 2.1 Inclusion and Exclusion Criteria

The inclusion criteria of this study were as follows: (a) Patients underwent transurethral surgery without planned ICU admission. (b) Age over 60 years old. (c) Body mass index (BMI) of 18 ~ 30 kg/m^2^. (d) The score on the American Society of Anesthesiologists Physical Status (ASA‐PS) scale is Grade I ~ III. (e) Estimated operation time of more than 30 min. (f) Signed informed consent with an understanding of the difference between the trial groups.

The exclusion criteria of this study were as follows: (a) Patients with uncontrolled hypertension (systolic blood pressure > 160 mmHg), diabetes (blood sugar at any time > 11.1 mmol), and heart failure (< 6 months). (b) Patients with abnormal liver function, defined as AST and/or ALT > 2.5 × ULN (Upper Limit of Normal), TBIL > 1.5 × ULN. And patients with abnormal renal function, defined as urea nitrogen > 1.5 × ULN, and blood creatinine > ULN. (c) Patients with preoperative cognitive impairment or a history of taking psychotropic drugs. (d) Patients allergic to benzodiazepines, propofol, muscle relaxants, and opioids.

Participants were excluded for the following reasons: (a) request for withdrawal. (b) breach of trial arrangements. (c) conversion to open laparotomy or endoscopy surgery. For follow‐up failure instances, researchers should make every effort to supplement the evaluation items that can be obtained.

### 2.2 Ethical Statement

The study protocol received approval from the Ethics Committee of the Affiliated Lianyungang Hospital of Xuzhou Medical University (approval number: KY20200328001). The trial was carried out in compliance with the Declaration of Helsinki and was registered at the Chinese Clinical Trial Registry (ChiCTR2000031798) before participant enrollment. After providing oral and written information for the study, written informed consents were obtained from the patients.

### 2.3 Study Procedures and Interventions

Before surgery, all patients were routinely required to fast for 8 h and abstain from drinking for 4 h. After getting into the operating room, the electrocardiogram (ECG), heart rate (HR), non‐invasive blood pressure (NIBP), and saturation of peripheral oxygen (SpO_2_) were monitored. The intraoperative hemodynamic changes were monitored in real‐time by catheterizing the radial artery, and the Bispectral Index (BIS) was employed to monitor the depth of anesthesia. Sufentanil 0.3 µg/kg, trial sedatives, and cisatracurium 0.15 mg/kg were gently delivered after pre‐oxygenation for 5 min. The laryngeal mask was recommended as a ventilation tool over tracheal intubation [3]. Endotracheal intubation was selected for patients with poor laryngeal mask position or great tooth motion. Typically, 3 ~ 5 min after treatment, a laryngeal mask or an endotracheal intubation was carried out. Propofol was administered intravenously to patients in Group P at a maintenance rate of 4 ~ 10 mg/kg/h after receiving an induction dose of 1–2 mg/kg. Patients in Group R were given remimazolam tosylate 0.2–0.3 mg/kg as an induction dose, and remimazolam was pumped at a rate of 0.5–1 mg/kg/h intraoperatively. The anesthesiologist adjusted the infusion rate and dose of sedative drugs according to clinical judgment to maintain the BIS value between 40 and 60. The maintenance rate of remifentanil was 0.05–0.1 µg/kg/min intraoperatively. Due to the short procedure time, additional intraoperative muscle relaxants were generally not required. And if necessary, additions were at the discretion of the attending anesthesiologist.

For the problem of relatively high BIS during the induction, remimazolam 0.05 mg/kg and propofol 0.5 mg/kg were allowed to be added accordingly. The number of additions less than or equal to twice was allowed; otherwise, it was considered a failure of anesthesia‐induced sedation. When the blood pressure dropped to 30% of the baseline value, norepinephrine 8 μg was given, and atropine 0.5 mg was administered when the heart rate dropped to 45 beats per minute. The infusion volume was adjusted in accordance with the patient's vital signs, the intraoperative circulatory status, and the length of the procedure. The changes in blood pressure, HR, and BIS value were accurately recorded at several different time points: after admission (T_0_), after administration(T_1_), time at inserting laryngeal mask or tracheal tube (T_2_), time at the placement of urethroscope (T_3_), 30 min after the operation (T_4_), end of the operation (T_5_).

Given that there were great individual differences in the appropriate remimazolam concentration during anesthesia maintenance and the awakening time was a little long after stopping the infusion of remimazolam in some patients in the pre‐experiment, we decided that the remimazolam group was routinely given 0.5 mg flumazenil for antagonism and the patients were sent to the recovery room for further observation until they awoke.

During the preoperative visit and postoperative follow‐up, 3 mL of arterial blood was obtained from the patient. ELISA was used to measure serum levels of IL‐6 and TNF‐ after the blood was centrifuged. The Chinese version of the QoR‐15 questionnaire was utilized to evaluate the quality of postoperative functional recovery on the day prior to surgery and the first postoperative day.

### 2.4 Index of Observations

The primary outcome was the occurrence of hypotension during induction and maintenance of anesthesia. Hypotension was defined as blood pressure that was less than 70% of the preoperative baseline value for 1 min and necessitated the use of a continuous intraoperative pump or several intravenous bolus vasopressors. The secondary outcome measures included intraoperative haemodynamic indices, the quality of recovery after anesthesia, levels of inflammatory factors before and after surgery, time to leave the PACU, and the incidence of adverse events. When Aldretes score exceeded 9, patients were sent out of the PACU. The successful perioperative sedation was defined as the absence of (1) intraoperative awareness and movement, (2) the need for other sedatives. Before the patient left the PACU, the incidence of nausea and vomiting, hypoxemia, lethargy, and delirium was noted. Patients in the PACU were evaluated for delirium using the nursing delirium assessment Scale. The occurrence of intraoperative awareness was investigated using a modified Brice questionnaire.

### 2.5 Statistical Analysis

The sample size calculation of this study referred to the study by Hirata, assuming that the remimazolam group can reduce the incidence of hypotension by 27% compared with the propofol group. Accounting for a dropout rate of 10%, a sample size of 80 would be sufficient to attain 80% power with a type‐I error probability set at 0.05 [4].

The data were analyzed by the SPSS version 25.0(IBM Corporation) statistical software. The Kolmogorov–Smirnov test was used to test the normality of continuous variables. Mean and standard deviations (SD) were employed to express the normally distributed quantitative data. Non‐normally distributed data were described on median (interquartile). Categorical variables are described in numbers (percentage). Repeated‐measures analysis of variance was carried out for different time points within the group. A nonparametric test was used for measurement data that failed to follow a normal distribution. Binary logistic regression was used to analyze the risk factors of intraoperative hypotension. Multiple linear regression was used to analyze the effect of data recorded during surgery on the time out of PACU. *p* < 0.05 was considered a statistically significant difference.

## 3 Results

### 3.1 Baseline Characteristics

**Figure 1:**
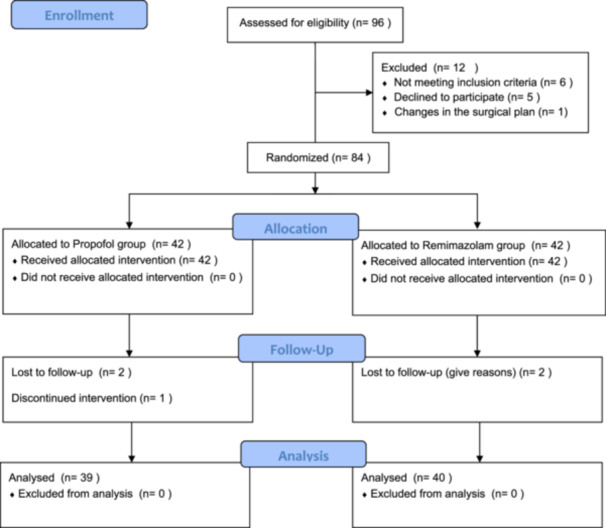
The flow diagram of study recruitment and analysis.

We initially evaluated 96 patients and finally analyzed 79 patients by Intention‐To‐Treat. The flow diagram of study recruitment and analysis was illustrated in Figure 1. There were no significant differences in age, BMI, ASA classification, anesthetic duration, and preoperative baseline vital signs between Group P and Group R (*p *> 0.05; Table 1).

**Table 1:** Baseline characteristics.

Characteristics	Group R	Group P	*p*
Age (years)	67.95 ± 8.31	67.13 ± 8.65	0.668
Sex (male/female)	31/9	28/11	0.560
BMI (kg m^−2^)	24.97 ± 3.19	24.58 ± 2.84	0.574
ASA (II/III)	33/7	32/7	0.958
Comorbidities (yes/no)			
Hypertension	15/25	19/20	0.314
Diabetes	5/35	5/34	1.000
Type of surgery			0.433
Bladder lesion resection	8	9	
Ureteral lithotripsy	9	13	
Vaporresection of prostate	23	17	
Intubation/laryngeal mask	17/23	22/17	0.216
Anesthetic duration (min)	67.50 [48.25–94.50]	74.00 [58.00–107.00]	0.124
Duration of surgery (min)	47.25 [33.78–66.15]	51.80 [40.60–74.90]	0.138
Systolic pressure (mmHg)	158.08 ± 20.29	163.3 ± 15.19	0.199
Diastolic pressure (mmHg)	76.15 ± 9.92	79.44 ± 10.57	0.158
Pulse (min)	72.63 ± 10.67	71.54 ± 12.13	0.673
Preoperative QoR‐15 scores	141.5 (8.8)	141.0 (6.0)	0.859

### 3.2 Vital Signs

**Figure 2:**
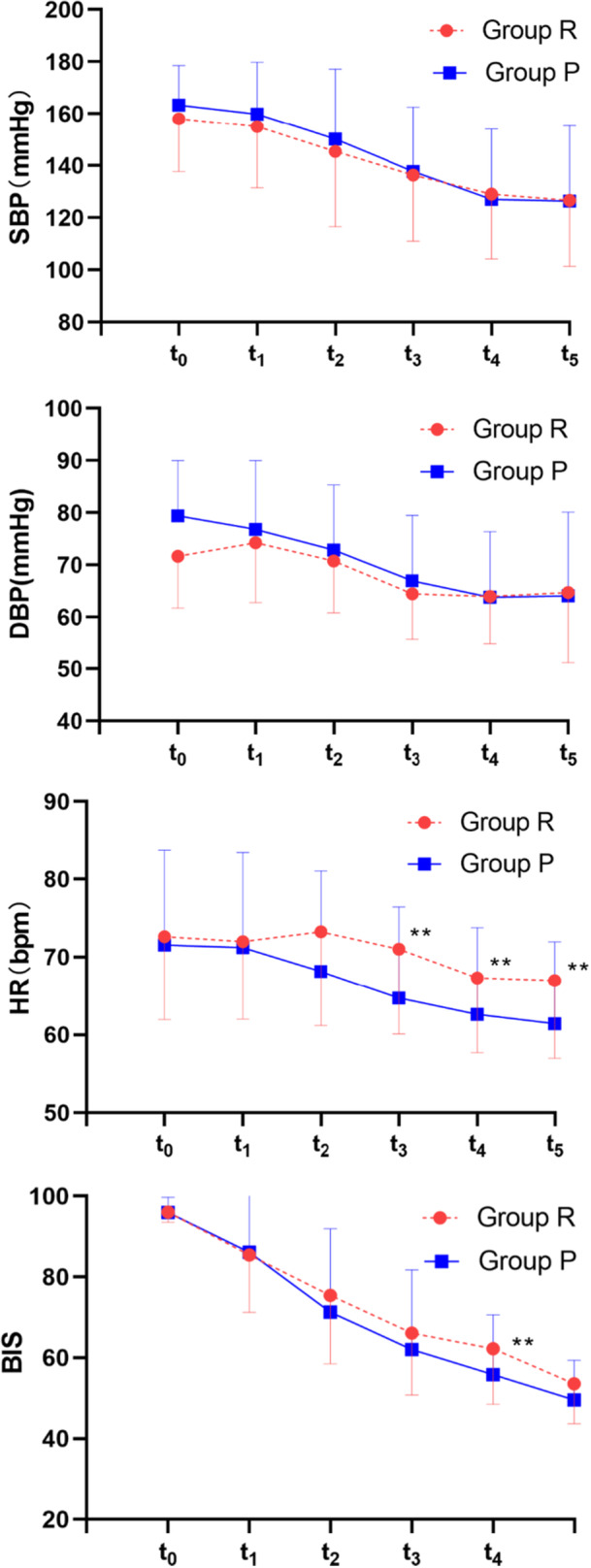
The mean and standard deviation of SBP, DBP, HR, and BIS during induction.

**Figure 3:**
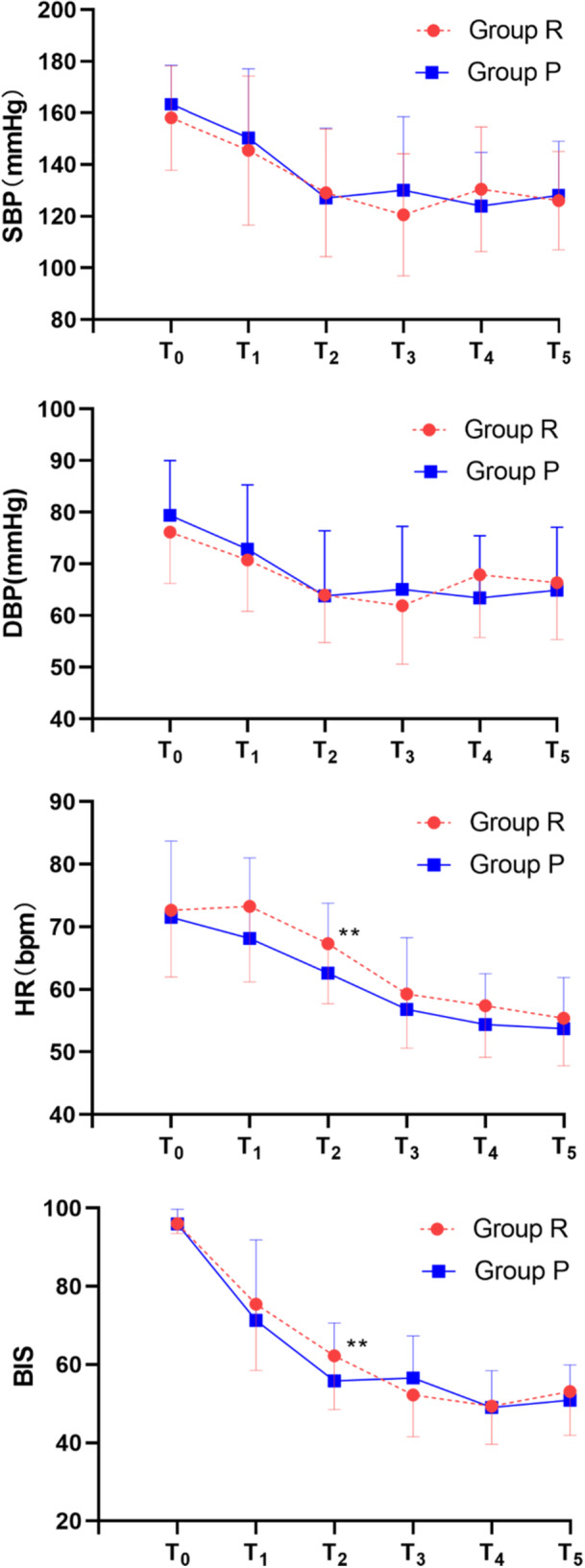
The mean and standard deviation of SBP, DBP, HR, and BIS measured at different time points throughout the anesthesia.

There was no discernible difference between the group and time point crossover effects and the pure time point impact in the blood pressure of the two groups within 5 min after anesthetic induction (Figure 3). The mean and standard deviation of SBP, DBP, HR, and BIS during induction and at different time points throughout the anesthesia were shown in Figures 2 and 3. When the urethroscopic device was placed, the blood pressure in Group R was noticeably lower than that of Group P (Figure 3), and it was somewhat higher during the operation. It demonstrated how remimazolam might more effectively maintain hemodynamic stability while fending off adverse surgical stimuli. Induction hypotension rather than intraoperative hypotension made up the majority of the variation in the incidence of hypotension between the two groups. The general data of the intraoperative hypotensive group and non‐hypotensive groups were statistically analyzed, and the results showed that there were differences in preoperative chronic hypertension, duration of anesthesia, and total remifentanil dosage in the two groups (*p *< 0.05; Table 2). The above factors were introduced into the model as independent variables for binary logistic regression analysis, and the results showed that preoperative chronic hypotension was an independent risk factor for intraoperative hypotension. Multivariate analysis of intraoperative hypotension for regression analysis was shown (*p *< 0.05; Table 3). The heart rate of patients in Group R was higher than in Group P throughout the anesthesia period, and there was a statistical difference at 3 ~ 5 min after administration (*p* = 0.015, *p* = 0.048, *p* = 0.018, respectively) and inserting a laryngeal mask or endotracheal tube(*p* = 0.028). The BIS value showed a trend of steep decline during the induction period. There was a difference in the BIS value between the two groups at 4 min after anesthesia, that is, when the ventilation tool was placed (*p *= 0.049). The levels of IL‐6 and TNF‐α increased postoperatively. Tables 4 and 5 provide a comparison of some indicators in blood gas analysis and levels of inflammatory factors.

**Table 2:** Univariate analysis of intraoperative hypotension for regression analysis.

Variable	Intraoperative hypotension		*p* value
	Present (*n* = 26) Absent (*n* = 53)		
Age (year)	66.85 ± 8.23	67.89 ± 8.59	0.609
BMI (kg m^−2^)	25.16 ± 3.09	24.59 ± 2.98	0.429
Chronic hypertension (yes/no)	17/9	17/36	0.005*
Diabetes (yes/no)	4/22	6/47	0.722
Coronary heart disease (yes/no)	2/24	2/51	0.841
Tracheal tube/laryngeal mask	11/15	29/24	0.300
Remimazolam/Propofol	16/10	30/23	0.130
Baseline SBP (mmHg)	157.23 ± 14.98	162.34 ± 19.27	0.239
Anesthetic duration (min)	65.38 ± 26.76	86.19 ± 39.88	0.019*
Total remifentanil (µg)	340.73 ± 183.39	491.14 ± 306.99	0.024*
Total fluid (mL)	853.85 ± 360.53	908.58 ± 352.29	0.521

Abbreviation: SBP, systolic blood pressure.

* *p* < 0.05 between intraoperative hypotension and absence of intraoperative hypotension.

**Table 3:** Multivariate analysis of intraoperative hypotension for regression analysis.

Variable	OR (95%CI)	*p* value
Remimazolam/Propofol	2.506 (0.804–7.807)	0.113
Chronic arterial hypertension	5.187 (1.651–16.297)	0.005*
Anesthetic duration	0.987 (0.955–1.019)	0.416
Total remifentanil	0.998 (0.993–1.002)	0.346

Abbreviations: CI, confidence interval; OR, odds ratio.

* *p* < 0.05 between intraoperative hypotension and absence of intraoperative hypotension.

**Table 4:** Comparison of Glu and Lac before and after surgery.

Group	Glu		Lac	
	Preoperative postoperative		Preoperative postoperative	
Group R	6.25 ± 1.57	6.35 ± 1.62	0.98 ± 0.32	0.76 ± 0.40
Group P	6.06 ± 1.42	5.98 ± 1.35	1.08 ± 0.45	0.95 ± 0.36
*p* value	0.578	0.273	0.274	0.030*

* *p* < 0.05 between Group R and Group P.

**Table 5:** Comparison of IL‐6 and TNF‐α before and after surgery.

Group	IL‐6		TNF‐α	
	Preoperative postoperative		Preoperative postoperative	
Group R	17.33 ± 1.05	19.49 ± 1.02	2.29 ± 1.26	11.16 ± 0.76
Group P	16.69 ± 2.05	17.91 ± 1.81	2.68 ± 1.38	11.56 ± 1.02
*p* value	0.085	0.076	0.203	0.053

* *p* < 0.05 between Group R and Group P.

### 3.3 Anesthesia Awakening and Recovery

**Figure 4:**
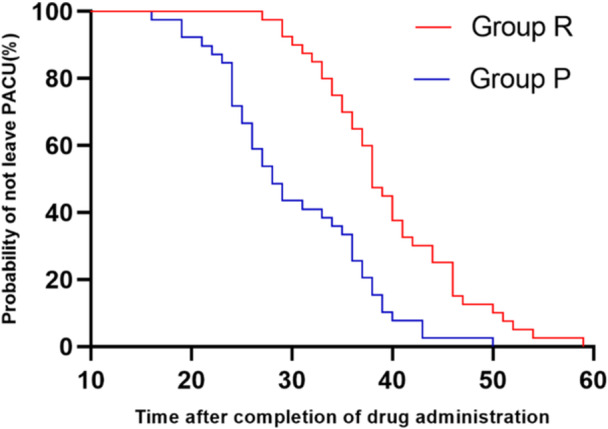
Kaplan–Meier curve from completion of trial drug administration to the time when patients were able to leave the PACU.

The average time from discontinuation of drug administration to extubation of the patients in Group R and Group P were (2.67 ± 0.93) min and (7.00 ± 1.38) min, respectively (*p* < 0.05). The time from the end of the final drug administration to the discharge criteria of the patients in Group R and Group P were (39.65 ± 7.33) min and (30.28 ± 7.81) min (*p *< 0.05; Table 6). The completion of trial drug administration to the time when patients were able to leave the PACU is shown in Figure 4. On the first postoperative day, the QoR‐15 scores in the remimazolam group were 9 points lower than those in the propofol group, reflected in physical comfort and emotional state. Statistical analysis of the correlation between each of the study variables and the length of PACU stay revealed that gender, type of sedative, age, ASA classification, and the maintenance rate of remifentanil were associated with PACU residence time (*p *< 0.05; Table 7). The above factors were again included in the multiple linear regression model to obtain the regression equation (PACU residence time = 6.738 − 7.046*type of sedative + 0.442*age, *R* ^2 ^= 0.587; Table 8).

**Table 6:** Intraoperative condition and postoperative recovery.

	Group R	Group P	*p*
Hypotension (none/PIH/IOH/PIH + IOH)	20/10/4/6	12/11/11/5	0.145
Duration of hypotension (min)	0 [0,10]	7.5 [1.24,25]	0.008*
Success sedation (yes/no)	37/3	38/1	0.626
Total propofol (mg)		500.00 [395.00–735.00]	
Total remimazolam (mg)	69.00 [52.25–90.00]		
Total remifentanil (µg)	409.65 ± 219.09	474.44 ± 331.71	0.308
Total norepinephrine(µg)	0.0 [0.00–16.00]	24.50 [0.00–80.00]	0.000*
Atropine (yes/no)	2/38	6/33	0.247
Intraoperative fluid (mL)	858.68 ± 372.97	938.51 ± 337.51	0.335
Time to extubation (min)	2.67 ± 0.93	7.00 ± 1.38	0.000*
Time to leave PACU (min)	39.65 ± 7.33	30.28 ± 7.81	0.000*
Postoperative QoR‐15 scores	115.0 (9.0)	124.0 (6.0)	0.000*

Abbreviations: IOH, intraoperative hypotension; PACU, postanesthesia care unit; PIH, post‐induction hypotension.

* *p* < 0.05 between Group R and Group P.

**Table 7:** Correlation analysis between PACU residence time and study variables.

Variable	Length of stay in PACU correlation coefficient (R‐value)	*p* value
Remimazolam/Propofol	−0.531	0.000*
Male/female	−0.586	0.000*
Age	0.532	0.000*
ASA classification	0.335	0.003*
Tracheal tube/laryngeal mask	0.007	0.950
The duration of hypotension	−0.160	0.160
The duration of BIS < 30	0.105	0.357
The duration of 30 < BIS < 40	−0.262	0.019*
Anesthetic duration	0.056	0.627
The maintenance rate of remifentanil	0.230	0.042*

Abbreviation: PACU, post‐anesthesia care unit.

* *p* < 0.05 means the correlation.

**Table 8:** Multiple linear regression analysis of PACU residence time.

Model	Unstandardized coefficient		*t*	Sig
	B SD			
Constant	6.738	6.608	1.020	0.311
Remimazolam/Propofol	−7.046	2.209	−3.189	0.002*
Male/female	−1.427	1.714	−0.832	0.408
Age	0.442	0.094	4.673	0.000*
The duration of 30 ＜ BIS ＜ 40	−0.038	0.08	−0.477	0.635
The maintenance rate of remifentanil	43.619	25.349	1.721	0.090

Abbreviation: SD, standard deviation.

* *p* < 0.05 means significant correlation.

### 3.4 Adverse Events

**Figure 5:**
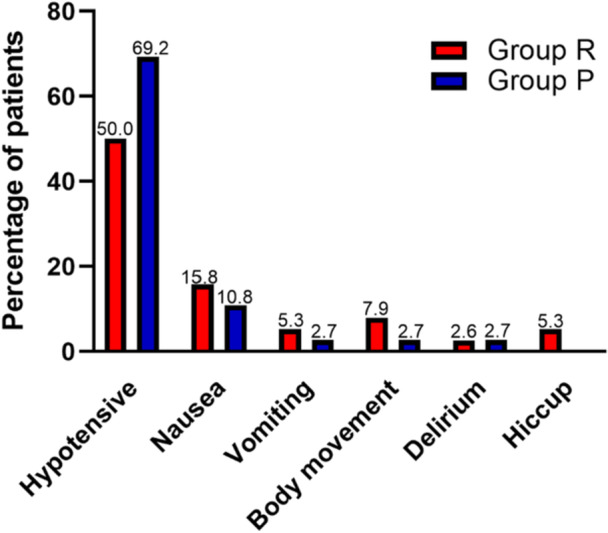
Comparison of the incidence of adverse reactions between Group R and Group P.

No life‐threatening serious adverse events occurred in either group throughout the trial. The rate of hypotension after administration was 69.2% in the propofol group and 50% in the remimazolam group (*p * < 0.05; Figure 5). Duration of hypotension was lower in the remimazolam group (0 [0,10] vs. 7.5 [1.24, 25] min, *p* = 0.008). The incidence rate of injection pain was 2.5% in the remimazolam group, which was lower than 10.3% in the propofol group. Hiccups occurred in 5.3% of patients in Group R, but it did not occur in Group P.

## 4 Discussion

Compared with traditional open or laparoscopic surgery, natural orifice transluminal endoscopic surgery has the advantage of less trauma, faster postoperative recovery, and higher patient satisfaction [5]. Transurethral surgery is a common surgical procedure in urology, such as prostate resection, bladder tumor resection, and ureteral lithotripsy. Effective sedation and analgesia can relieve the patient's physical discomfort and provide good operating conditions for the surgeon.

Propofol, the most commonly used intravenous anesthetic, is highly lipophilic and can rapidly cross the blood–brain barrier to achieve deep sedation in a short period. However, in clinical application of propofol sedation, a variety of cardiopulmonary complications frequently emerge. These include hypotension, arrhythmia, respiratory depression, and injection‐site pain as well [6]. Remimazolam tosylate, a novel benzodiazepine sedative, has a short elimination half‐life, and its metabolism is not reliant on cellular P450 enzyme activity. When compared to comparable anesthetics, remimazolam exhibits a more rapid onset of action and metabolism, and its metabolites lack biological activity [6]. Remimazolam has good efficacy and safety for clinical treatment [7] and diagnostic procedure sedation [8].

The incidence rate of hypotension in Group R was significantly lower than that in Group P. The difference in incidence was mainly reflected in intraoperative hypotension rather than post‐induction hypotension, indicating that remimazolam had less effect on circulation inhibition. A recent study has shown that in moyamoya disease surgeries requiring strict blood pressure management, such as cerebral revascularization, the hemodynamic stability of total intravenous anesthesia with remimazolam is superior to that of anesthesia induced by propofol and maintained with desflurane [9].

The overall fluctuation range of blood pressure and the heart rate in Group R was smaller compared with Group P, probably owing to the differential effect of remimazolam and propofol on autonomic nervous activity. Hasegawa found that the change of the normalized unit of low frequency power, which reflects both sympathetic and parasympathetic activities before and after anesthesia, was less in the remimazolam group than in the propofol group, and propofol broke the balance of sympathetic and parasympathetic activities and modulated it to a sympathetic dominance [10]. The BIS was relatively high during general anesthesia with remimazolam. Correspondingly, we can observe an increase in β waves on the electroencephalogram. Shirozu et al. used several different indications, including SEF of Sedline and pupillary diameter, as a supportive indicator to confirm sedation level during remimazolam anesthesia [11]. To prevent the occurrence of intraoperative awareness due to insufficient sedation, we used the modified Brice questionnaire in the PACU to investigate whether it had occurred [12].

No serious adverse events occurred in all patients during the recovery period and 24 h after the operation. The incidence of nausea and vomiting in Group P was 10.8% and 2.7%, which was lower than in Group R 15.8% and 5.3%. The difference may be attributed to the antiemetic effect of propofol and the use of anticholinergic atropine due to bradycardia in the propofol group [13]. The patients in the propofol group could cause intraoperative dreams, but there were no intraoperative dreams in the remimazolam group [14]. It may be that propofol activates the choline neurons of the basal forebrain to promote the occurrence of dreams [14]. Studies have shown that changes in the inflammatory response and neurological injury‐related biomarkers were associated with the occurrence and duration of the emergence of delirium in elderly patients, and IL‐6 had a significant correlation with the duration of emergence delirium [15]. In this study, the concentrations of IL‐6 and TNF‐α in both groups were elevated after surgery, suggesting that the inflammatory response in patients was activated. However, there was no difference in the incidence of delirium. Two patients who received remimazolam occurred hiccups, of whom one relieved spontaneously for about 10 s and the other lasted for about 3 min. Zhang et al. [16] reported a similar incidence of hiccups in hysteroscopy, whereas Huang et al. [17] found the probability of hiccups was higher than 10% after remimazolam 0.3 mg/kg bolus. The reason for the apparent discrepancy probably lies in the use of sufentanil before the induction of remimazolam and cisatracurium immediately after remimazolam.

The level of consciousness in Group R recovered rapidly after flumazenil antagonism. This result was consistent with the findings of Worthington et al., that the sedation was rapidly reversible 1 min after the use of flumazenil [18]. However, the recovery time was longer than that in the propofol group (*p* ＜ 0.05), which may be related to the re‐sedation caused by remimazolam [19]. The plasma protein binding of remimazolam was 92%, which was higher than that of flumazenil (31.4%). The affinity constant of remimazolam for the benzodiazepine receptor was 0.013 µg/mL compared to the affinity constant of flumazenil (2.7 ng/mL). As the blood concentration of flumazenil decreases, the sedative effect of remimazolam may reappear as the duration of action of remimazolam is similar to that of flumazenil. Chen et al. also recommended that the residual effect of remimazolam after continuous infusion needed a further psychomotor assessment before hospital discharge [20]. Similar to the study by Mao et al. we found that patients in Group R had lower QoR‐15 scores than those in the propofol group on the first postoperative day, possibly due to the effect of benzodiazepines on cognition and the rewarding effects of propofol [21].

There are some limitations in this study. First, the patients involved in this study were all from a single center, and further multi‐center research and analysis are needed. Second, patients in the remimazolam group lacked continuous observation and assessment of neurophysiological function and cognition after flumazenil antagonism. Third, remimazolam plasma concentration was not monitored after the surgery to prevent adverse outcomes from re‐sedation. Fourth, we did not follow up with patients for 3 days or more in postoperative recovery.

## 5 Conclusion

Both remimazolam and propofol can provide safe and effective sedation for elderly patients undergoing transurethral surgery. Remimazolam can largely avoid severe blood flow fluctuation, deep sedation, and injection pain, but the moderate residual effect of remimazolam on neurophysiological function needs further care and evaluation. In the meantime, we need to pay attention to the short‐term changes in the physical comfort and emotional state in terms of the quality of post‐operative recovery of remimazolam. In conclusion, remimazolam tosylate can be safely used as an alternative to propofol for anesthesia in elderly urological patients.

## Author Contributions

**Zhi Cheng:** writing – original draft, writing – review and editing, data curation, software, validation, visualization. **Jiachi Li:** software, data curation, and formal analysis. **Ying Zhang:** data curation and project administration. **Guangrong Dai:** conceptualization, methodology, investigation, supervision, funding acquisition, and resources.

## Conflicts of Interest

The authors declare no conflicts of interest.

## Transparency Statement

The corresponding author, Guangrong Dai, affirms that this manuscript is an honest, accurate, and transparent account of the study being reported; that no important aspects of the study have been omitted; and that any discrepancies from the study as planned (and, if relevant, registered) have been explained.

## Data Availability

The data that support the findings of this study are available from the corresponding author on request. The data are not publicly available due to privacy or ethical restrictions.
